# Association between hyperglycemia and adverse clinical outcomes of sepsis patients with diabetes

**DOI:** 10.3389/fendo.2022.1046736

**Published:** 2023-01-09

**Authors:** Shan Lin, Dingfeng Lai, Wanmei He

**Affiliations:** ^1^ Department of Respiratory and Critical Care Medicine, Affiliated Hospital of North Sichuan Medical College, Nanchong, Sichuan, China; ^2^ Department of Medical Intensive Care Unit, The First Affiliated Hospital of Sun Yat-sen University, Guangzhou, Guangdong, China

**Keywords:** critical care, diabetes, sepsis, glucose, prognosis

## Abstract

**Background:**

Hyperglycemia is one of the poor prognostic factors in critical ill sepsis patients with diabetes. We aimed to assess the interaction between admission glucose level and clinical endpoints in sepsis patients with diabetes admitted in the intensive care unit (ICU).

**Methods:**

Data from the Medical Information Mart Intensive Care III database were used in this study. The study primary endpoint was 28-day mortality after ICU admission. Multivariate Cox regression models were used to explore the association between admission glucose level and the primary endpoint.

**Results:**

We included 3,500 sepsis patients with diabetes. Of participants with no hyperglycemia, mild hyperglycemia, and severe hyperglycemia, no differences were evident in hospital mortality, ICU mortality, or 28-day mortality (all *P >*0.05). The multivariable Cox regression analysis demonstrated that severe hyperglycemia did not increase the risk of 28-day mortality (hazard ratio [HR]=1.06, 95% confidence interval [CI]: 0.86–1.31, *P*=0.5880). Threshold effects analysis identified the inflection points for 28-day mortality as 110 mg/dl and 240 mg/dl. The HRs for 28-day mortality were 0.980 in the <110 mg/dl and 1.008 in the >240 mg/dl. A short-term survival advantage was observed in the 110–240 mg/dl group compared with that in the <110 mg/dl group; meanwhile, no adverse hazard was detected in the >240 mg/dl group. In the stratified analyses, the association effect between the three glucose groups (<110 mg/dl, 110–240 mg/dl, and ≥240 mg/dl) and 28-day mortality was consistent in terms of different sequential organ failure assessment (SOFA) scores and infection sites. The 28-day mortality of the 110–240 mg/dl group with a SOFA score of ≥10 was lower than that of the <110 mg/dl group (HR=0.61, 95% CI: 0.38–0.98).

**Conclusion:**

Admission hyperglycemia was not a risk factor for short-term prognosis in critical ill sepsis patients with diabetes; a lower admission blood glucose level was associated with increased risk of poor prognosis. The potential benefit of higher admission glucose level on 28-day mortality in patients with a more severe condition remains a concern.

## Background

Diabetes is a common comorbidity among critically ill sepsis patients and generally causes immune dysfunction and metabolic disorders, including hyperglycemia (1–3). In recent years, diabetes is developing swiftly as a global health epidemic and is one of the top ten causes of adult death (4). Hyperglycemia was closely related to endothelial cell injury, mitochondrial damage, and inflammation activation (5, 6). In terms of clinical research, Vught et al. revealed that severe hypoglycemia contributed to higher 90-day mortality in sepsis patients with diabetes (7). In another study, Vught et al. indicated that severe hyperglycemia was correlated with 30-day mortality in patients with sepsis, regardless of the presence or absence of diabetes (8). Subsequently, multiple studies that examined the glucose levels of this patient group reported different views, and some indicated the adverse effects of glycemic control (9–12). A previous large randomized trial found that a glucose level of 81–108 mg/dl was associated with adverse clinical outcomes of glycemic control compared with a glucose level of ≤180 mg/dl (2).

To our knowledge, evidence on how hyperglycemia affects the clinical outcomes in critical sepsis patients with diabetes remains limited and debatable. Considering that diabetes is consistently correlated with other diseases, the impact of admission glucose level in the outcome of sepsis patients should be explored, potentially determining better individualized glycemic control strategies. Consequently, we aimed to assess the interaction between admission glucose levels and clinical endpoints in sepsis patients with diabetes admitted in the intensive care unit (ICU).

## Methods

### Patient data

Data from the Medical Information Mart Intensive Care III (MIMIC-III) database were used in this study (13). The institutional review boards of Beth Israel Deaconess Medical Center and Massachusetts Institute of Technology Affiliates approved the access to the database (record identification numbers: 33460949 and 49780033). The requirement for obtaining informed consent was waived due to the use of anonymized data.

Adult (aged ≥18 years) patients diagnosed with sepsis based on the following criteria were included in the study: suspected infection and a sequential organ failure assessment (SOFA) score of ≥2 (14). We excluded patients with 1) multiple ICU admissions, 2) less than one day of follow-up, 3) hospital length of stay less than the ICU length of stay, 4) no diabetes, and 5) admission blood glucose level of <70 mg/dl. The first plasma glucose measurement obtained in patients admitted in the ICU was used in the study and grouped into the following categories: no hyperglycemia (≤139 mg/dl), mild hyperglycemia (140–199 mg/dl), and severe hyperglycemia (≥200 mg/dl) (7, 8). Along with the patient’s baseline information (e.g., age and sex), therapeutic measures, and clinical endpoints for routine variables, we also extracted the data of patients’ SOFA score, Elixhauser Comorbidity Index (SID30) (15), and specific comorbidities. The code for assisting in the investigation of MIMIC-III is openly available on the website (16).

### Outcomes

The primary outcome was 28-day mortality after ICU admission, and the secondary outcome was ICU mortality.

### Statistical analysis

The data were expressed as mean ± standard deviation or median (interquartile range) for continuous variables and as numbers and percentages for categorical variables. We compared the characteristics of participants between glucose groups using one-way analysis of variance for continuous variables and chi-square test for categorical variables. Initially, we applied Cox regression models to explore the associations of admission glucose level with the 28-day mortality and logistic regression models to explore the association of admission glucose level with ICU mortality. We presented different adjusted models to assess the effect of admission glucose level on clinical endpoints in sepsis patients with diabetes. In model I, we adjusted for demographic characteristics (age and sex), disease severity (SOFA scores), comorbidity scores (SID30), infection site, and initial treatment (mechanical ventilation and renal replacement therapy on the first); in model II, we substituted the SID30 with the specific diseases (congestive heart failure, cardiac arrhythmias, etc.). Covariate screening was used to include covariates as potential confounders if they changed the estimates of admission glucose level on 28-day mortality by more than 10% or were associated significantly with 28-day mortality.

Subsequently, to explore whether a nonlinear relationship exists between glucose level and 28-day mortality, we performed the smoothed spline method using a Cox model to fit the 28-day mortality (generalized additive model for fitting ICU mortality). If it existed, segmental regression models constructed during the threshold effects analysis were used to detect the inflection points, and the differences were compared by log-likelihood ratio tests (17). Next, the admission glucose level was re-grouped by inflection points, and the different adjustment models described above were used to evaluate the clinical outcome. Finally, stratified analysis and interaction tests were conducted to explore the consistency of the relationship between the inflection point grouping of glucose and 28-day mortality in the patient subgroups based on SOFA scores (<5, 5–10, and ≥10) and infection site. All data were analyzed using EmpowerStats (www.empowerstats.com) and R (http://www.R-project.org). A *P*-value of <0.05 was considered significant.

## Results

### Participants’ characteristics

**Figure 1:**
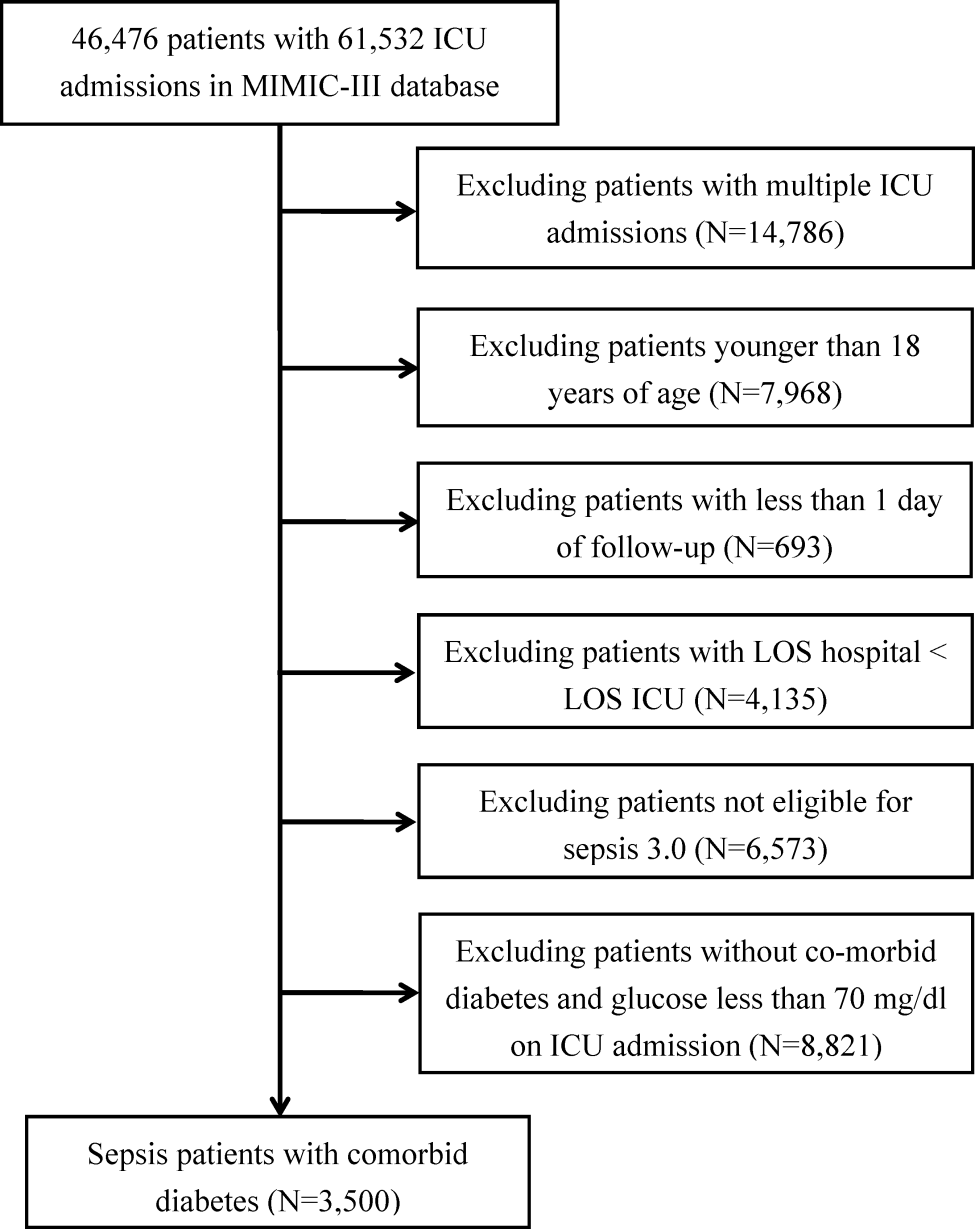
Flowchart of study participants. ICU, intensive care unit; LOS, length of stay.

A total of 3,500 sepsis patients with diabetes with a mean age of 66.79 years were enrolled in this study (**Figure 1**). Majority of the sepsis patients with diabetes were men (51.8% *vs.* 48.2%). No significant differences were observed between the three groups in terms of SID30, SOFA score, infection site, and need for mechanical ventilation or renal replacement therapy on the first day of ICU admission. Additional detailed results are presented in **Table 1**.

**Table 1:** Characteristics of participants.

Variables	All patients (N=3500)	No hyperglycemia (N=1271)	Mild hyperglycemia (N=1426)	Severe hyperglycemia (N=803)	*P*-value
Age (years)	66.8 ± 17.1	67.2 ± 16.6	66.1 ± 17.2	67.3 ± 17.3	0.176
Sex					0.387
Male	1814 (51.8%)	645 (50.7%)	759 (53.2%)	410 (51.1%)	
Female	1686 (48.2%)	626 (49.3%)	667 (46.8%)	393 (48.9%)	
Admission glucose (mg/dl)	165.4 ± 53.6	115.9 ± 17.2	166.0 ± 16.9	242.9 ± 41.0	<0.001
Infection site					0.629
Bloodstream	1507 (43.1%)	570 (44.8%)	596 (41.8%)	341 (42.5%)	
Pulmonary	238 (6.8%)	81 (6.4%)	102 (7.2%)	55 (6.8%)	
Abdominal	76 (2.2%)	25 (2.0%)	30 (2.1%)	21 (2.6%)	
Urinary tract	727 (20.8%)	258 (20.3%)	290 (20.3%)	179 (22.3%)	
Others	952 (27.2%)	337 (26.5%)	408 (28.6%)	207 (25.8%)	
Mechanical ventilation on first day	1746 (49.9%)	612 (48.2%)	729 (51.1%)	405 (50.4%)	0.287
Renal replacement therapy on first day	172 (4.9%)	69 (5.4%)	69 (4.8%)	34 (4.2%)	0.465
SOFA	5.0 (3.0-7.0)	5.0 (3.0-7.0)	5.0 (3.0-7.0)	5.0 (3.0-7.0)	0.280
Elixhauser Comorbidity index (SID30)	17.0 (8.0-26.0)	17.0 (8.0-26.0)	16.0 (8.0-25.0)	17.0 (9.0-26.0)	0.219
Length of ICU stay (days)	3.3 (1.8-7.8)	3.3 (1.8-7.9)	3.3 (1.8-7.8)	3.3 (1.8-7.8)	0.271
Length of hospital stay (days)	10.7 (6.3-18.5)	10.8 (6.3-19.1)	10.8 (6.2-18.0)	10.4 (6.2-18.4)	0.264
28-day mortality, n(%)	604 (17.3%)	226 (17.8%)	237 (16.6%)	141 (17.6%)	0.704
ICU mortality, n(%)	326 (9.3%)	105 (8.3%)	139 (9.8%)	82 (10.2%)	0.253
Hospital mortality, n(%)	520 (14.9%)	183 (14.4%)	213 (14.9%)	124 (15.4%)	0.804
Comorbidities, n(%)					
Congestive heart failure	1529 (43.7%)	585 (46.0%)	629 (44.1%)	315 (39.2%)	0.009
Cardiac arrhythmias	1327 (37.9%)	522 (41.1%)	535 (37.5%)	270 (33.6%)	0.003
Valvular disease	528 (15.1%)	240 (18.9%)	208 (14.6%)	80 (10.0%)	<0.001
Peripheral vascular disease	536 (15.3%)	237 (18.6%)	208 (14.6%)	91 (11.3%)	<0.001
Hypertension	2413 (68.9%)	896 (70.5%)	982 (68.9%)	535 (66.6%)	0.178
Other neurological diseases	409 (11.7%)	127 (10.0%)	178 (12.5%)	104 (13.0%)	0.059
Chronic pulmonary disease	805 (23.0%)	274 (21.6%)	334 (23.4%)	197 (24.5%)	0.259
Liver disease	357 (10.2%)	134 (10.5%)	134 (9.4%)	89 (11.1%)	0.396
Renal failure	1063 (30.4%)	425 (33.4%)	405 (28.4%)	233 (29.0%)	0.011
AIDS	17 (0.5%)	9 (0.7%)	4 (0.3%)	4 (0.5%)	0.280
Lymphoma	63 (1.8%)	21 (1.7%)	24 (1.7%)	18 (2.2%)	0.562
Metastatic cancer	163 (4.7%)	62 (4.9%)	75 (5.3%)	26 (3.2%)	0.084
Solid tumor	176 (5.0%)	56 (4.4%)	82 (5.8%)	38 (4.7%)	0.255
Obesity	398 (11.4%)	145 (11.4%)	157 (11.0%)	96 (12.0%)	0.795
Fluid and electrolyte disorders	1558 (44.5%)	548 (43.1%)	583 (40.9%)	427 (53.2%)	<0.001
Alcohol abuse	162 (4.6%)	66 (5.2%)	58 (4.1%)	38 (4.7%)	0.377
Drug abuse	60 (1.7%)	24 (1.9%)	20 (1.4%)	16 (2.0%)	0.492
Depression	305 (8.7%)	102 (8.0%)	125 (8.8%)	78 (9.7%)	0.412

ICU, intensive care unit; SOFA, sequential organ failure assessment; AIDS, acquired immune deficiency syndrome.

### Clinical outcomes of the participants

With regard to the clinical outcomes, the hospital mortality, ICU mortality, and 28-day mortality in sepsis patients with diabetes in the no hyperglycemia, mild hyperglycemia, and severe hyperglycemia groups were not significant (all *P >*0.05). No significant difference was found in the length of hospital or ICU stay among the three groups (all *P >*0.05).

### Associations between admission glucose level and clinical outcomes

The Cox regression analysis demonstrated that severe hyperglycemia did not increase the risk of 28-day mortality (crude hazard ratio [HR]=0.99, 95% confidence interval [CI] 0.80-1.22, *P* =0.9018). After adjusting for confounding factors, hyperglycemia remained a non-risk factor (**Table 2**). In model II, when compared with the no hyperglycemia group, the 28-day mortality rate in the severe hyperglycemia group did not significantly increase (HR=1.06, 95% CI: 0.86–1.31, *P*=0.5880). Similar findings were reported in the mild hyperglycemia group (HR=0.99, 95% CI: 0.82–1.19, *P*=0.9097). With regard to ICU mortality, the results similarly indicated no significant increase in ICU mortality in both the mild hyperglycemia and severe hyperglycemia groups compared with that of the no hyperglycemia group (**Table 2**).

**Table 2:** Association of admission glucose groups with primary and secondary outcomes.

28-day mortality	Groups	HR (95% CI)	*P*-value
Crude	No hyperglycemia	1.0	–
	Mild hyperglycemia	0.93 (0.78-1.12)	0.4346
	Severe hyperglycemia	0.99 (0.80-1.22)	0.9018
Model I	No hyperglycemia	1.0	–
	Mild hyperglycemia	0.98 (0.81-1.17)	0.7950
	Severe hyperglycemia	0.99 (0.81-1.23)	0.9625
Model II	No hyperglycemia	1.0	–
	Mild hyperglycemia	0.99 (0.82-1.19)	0.9097
	Severe hyperglycemia	1.06 (0.86-1.31)	0.5880
ICU mortality	Groups	OR (95% CI)	*P*-value
Crude	No hyperglycemia	1.0	–
	Mild hyperglycemia	1.20 (0.92-1.56)	0.1797
	Severe hyperglycemia	1.17 (0.85-1.61)	0.3346
Model I	No hyperglycemia	1.0	–
	Mild hyperglycemia	1.22 (0.94-1.60)	0.1412
	Severe hyperglycemia	1.16 (0.84-1.59)	0.3630
Model II	No hyperglycemia	1.0	–
	Mild hyperglycemia	1.24 (0.95-1.63)	0.1138
	Severe hyperglycemia	1.19 (0.87-1.65)	0.2805

Model I was adjusted by age, sex, SOFA, SID30, infection site, mechanical ventilation on first day, renal replacement therapy on first day.

Model II was adjusted by age, sex, SOFA, infection site, mechanical ventilation on first day, renal replacement therapy on first day, congestive heart failure, cardiac arrhythmias, valvular disease, peripheral vascular disease, hypertension, other neurological diseases, chronic pulmonary disease, liver disease, renal failure, AIDS, lymphoma, metastatic cancer, solid tumor, obesity, fluid and electrolyte disorders, alcohol abuse, drug abuse, and depression.

SOFA, sequential organ failure assessment; SID30, Elixhauser Comorbidity index; AIDS, acquired immune deficiency syndrome; HR, hazard ratio; OR, odds ratio; CI, confidence interval.

**Figure 2:**
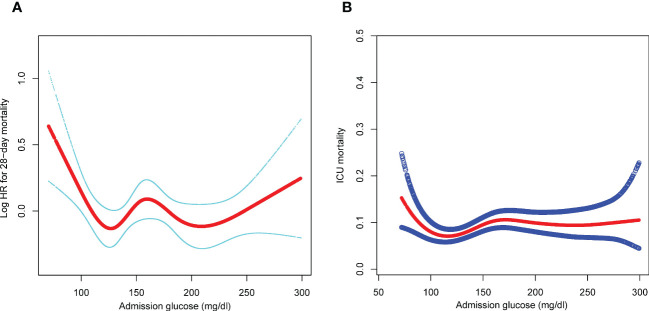
**(A)** Association of admission glucose level with 28-day mortality. **(B)** Association of admission glucose level with ICU mortality. adjusted by age, sex, SOFA, infection site, mechanical ventilation on first day, renal replacement therapy on first day, congestive heart failure, cardiac arrhythmias, valvular disease, peripheral vascular disease, hypertension, other neurological diseases, chronic pulmonary disease, liver disease, renal failure, AIDS, lymphoma, metastatic cancer, solid tumor, obesity, fluid and electrolyte disorders, alcohol abuse, drug abuse, and depression. SOFA, sequential organ failure assessment; AIDS, acquired immune deficiency syndrome; ICU, intensive care unit; HR, hazard ratio.

**Figure 3:**
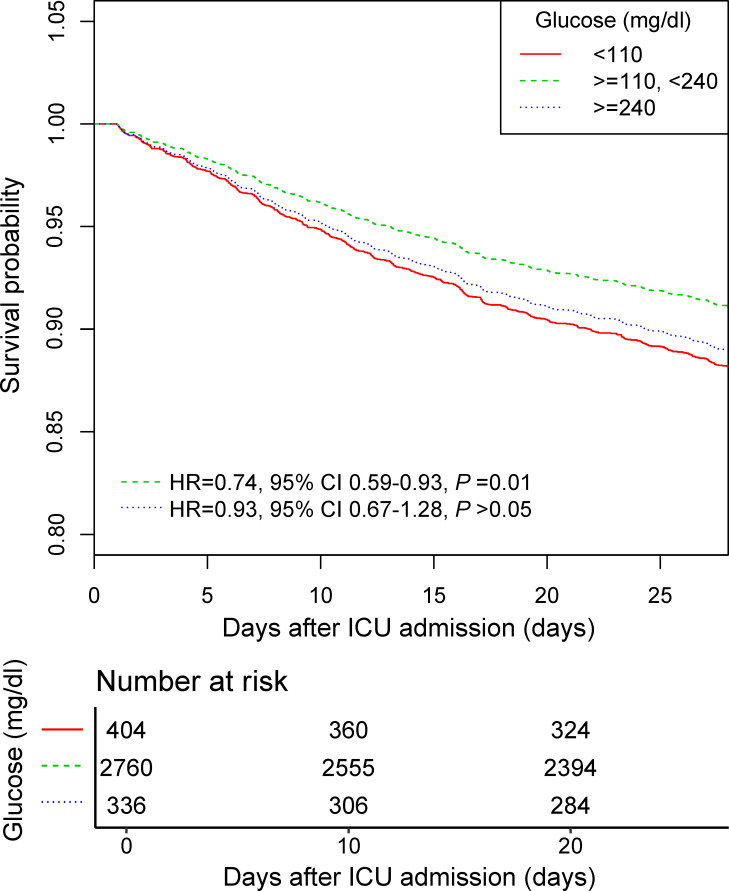
The 28-day survival curve of the Cox regression model for participants with inflection point grouping of glucose. Adjusted by age, sex, SOFA, infection site, mechanical ventilation on first day, renal replacement therapy on first day, congestive heart failure, cardiac arrhythmias, valvular disease, peripheral vascular disease, hypertension, other neurological diseases, chronic pulmonary disease, liver disease, renal failure, AIDS, lymphoma, metastatic cancer, solid tumor, obesity, fluid and electrolyte disorders, alcohol abuse, drug abuse, and depression. SOFA, sequential organ failure assessment; AIDS, acquired immune deficiency syndrome.

Smooth splines showed a nonlinear relationship of admission glucose with 28-day and ICU mortality (**Figures 2A, B**). Threshold effect analysis identified the inflection points for 28-day mortality of 110 mg/dl and 240 mg/dl. For 28-day mortality, the HR was 0.980 for a glucose level of <110 mg/dl and 1.008 for a glucose level of >240 mg/dl (**Table 3**). Subsequently, the admission glucose level was divided into three categories according to the inflection point: <110 mg/dl, 110–240 mg/dl, ≥240 mg/dl (inflection point grouping of glucose); a Cox regression analysis was performed, and the results revealed a 26% significant reduction of 28-day mortality in the 110–240 mg/dl group compared with the <110 mg/dl group (HR=0.74, 95% CI: 0.59–0.93, *P*=0.0100); in the >240 mg/dl group, no substantial increase was observed in the risk of 28-day mortality rate (*P >*0.05) (**Table 4**). A considerable short-term survival advantage was observed in the 110–240 mg/dl group compared with that in the 110 mg/dl group; meanwhile, no remarkable adverse hazard was detected in the >240 mg/dl group (**Figure 3**).

**Table 3:** Threshold effect analysis of glucose level and 28-day mortality rate using piece-wise linear regression.

Outcome: 28-day mortality			
Inflection point	HR	95% CI	*P*-value
< 110 mg/dl	0.980	0.968-0.990	0.0009
110-240 mg/dl	1.001	0.998-1.003	0.6563
> 240 mg/dl	1.008	1.002-1.013	0.0093

The log-likelihood ratio test: P <0.001

Adjusted by age, sex, SOFA, infection site, mechanical ventilation on first day, renal replacement therapy on first day, congestive heart failure, cardiac arrhythmias, valvular disease, peripheral vascular disease, hypertension, other neurological diseases, chronic pulmonary disease, liver disease, renal failure, AIDS, lymphoma, metastatic cancer, solid tumor, obesity, fluid and electrolyte disorders, alcohol abuse, drug abuse, and depression.

SOFA, sequential organ failure assessment; AIDS, acquired immune deficiency syndrome; HR, hazard ratio; CI, confidence interval.

**Table 4:** Associations between inflection point grouping of glucose and 28-day mortality.

28-day mortality	Groups	HR (95% CI)	*P*-value
Crude	<110	1.0	–
	≥110, <240	0.71 (0.56-0.89)	0.0029
	≥240	0.86 (0.62-1.18)	0.3430
Model I	<110	1.0	–
	≥110, <240	0.71 (0.57-0.89)	0.0033
	≥240	0.82 (0.60-1.14)	0.2380
Model II	<110	1.0	–
	≥110, <240	0.74 (0.59-0.93)	0.0100
	≥240	0.93 (0.67-1.28)	0.6492

Model I was adjusted by age, sex, SOFA, SID30, infection site, mechanical ventilation on first day, renal replacement therapy on first day.

Model II was adjusted by age, sex, SOFA, infection site, mechanical ventilation on first day, renal replacement therapy on first day, congestive heart failure, cardiac arrhythmias, valvular disease, peripheral vascular disease, hypertension, other neurological diseases, chronic pulmonary disease, liver disease, renal failure, AIDS, lymphoma, metastatic cancer, solid tumor, obesity, fluid and electrolyte disorders, alcohol abuse, drug abuse, and depression.

SOFA, sequential organ failure assessment; SID30, Elixhauser Comorbidity index; AIDS, acquired immune deficiency syndrome; HR, hazard ratio; CI, confidence interval.

In the stratified analysis, the association effect between the new glucose category and the risk of 28-day mortality was generally consistent in the different SOFA scores and infection site (**Table 5**). Furthermore, the 28-day mortality of the 110–240 mg/dl group with a SOFA score of ≥10 was lower than that of the <110 mg/dl group (HR=0.61, 95% CI: 0.38–0.98). Similarly, patients with bloodstream infection in the 110–240 mg/dl group experienced substantially lower 28-day mortality rate compared with those in the <110 mg/dl group (HR=0.70, 95% CI: 0.49–1.00).

**Table 5:** Association of inflection point grouping of glucose with 28-day mortality stratified by different scores of SOFA and infection site.

28-day mortality	Crude		Adjusted model		
Admission glucose (mg/dl)	HR (95% CI)	*P*-value	HR (95% CI)	*P*-value	*P* interaction
SOFA: <5					0.5759
<110	1.0	–	1.0	–	
≥110, <240	0.89 (0.59-1.34)	0.5809	0.91 (0.60-1.38)	0.6460	
≥240	1.21 (0.70-2.08)	0.4949	1.14 (0.65-1.99)	0.6445	
SOFA: ≥5, <10					
<110	1.0	–	1.0	–	
≥110, <240	0.69 (0.48-0.99)	0.0427	0.75 (0.52-1.09)	0.1284	
≥240	0.87 (0.54-1.42)	0.5832	1.03 (0.63-1.68)	0.9172	
SOFA: ≥10					
<110	1.0	–	1.0	–	
≥110, <240	0.54 (0.35-0.83)	0.0046	0.61 (0.38-0.98)	0.0399	
≥240	0.47 (0.22-1.01)	0.0521	0.55 (0.24-1.22)	0.1413	
Infection site: Bloodstream					0.7049
<110	1.0	–	1.0	–	
≥110, <240	0.75 (0.53-1.06)	0.1045	0.70 (0.49-1.00)	0.0474	
≥240	1.00 (0.62-1.62)	0.9977	1.05 (0.64-1.71)	0.8470	
Infection site: Pulmonary					
<110	1.0	–	1.0	–	
≥110, <240	0.39 (0.18-0.87)	0.0205	0.48 (0.21-1.08)	0.0776	
≥240	0.45 (0.13-1.48)	0.1873	0.47 (0.14-1.63)	0.2351	
Infection site: Abdominal					
<110	1.0	–	1.0	–	
≥110, <240	0.65 (0.21-1.95)	0.4396	0.54 (0.09-3.37)	0.5131	
≥240	1.41 (0.32-6.32)	0.6508	3.05 (0.18-52.01)	0.4410	
Infection site: Urinary tract					
<110	1.0	–	1.0	–	
≥110, <240	0.63 (0.38-1.03)	0.0650	0.65 (0.39-1.08)	0.0987	
≥240	0.62 (0.31-1.26)	0.1866	0.62 (0.30-1.27)	0.1892	
Infection site: Others					
<110	1.0	–	1.0	–	
≥110, <240	0.84 (0.52-1.36)	0.4809	0.93 (0.56-1.52)	0.7646	
≥240	0.98 (0.50-1.92)	0.9629	0.99 (0.50-1.99)	0.9834	

Adjusted by age, sex, SOFA, infection site, mechanical ventilation on first day, renal replacement therapy on first day, congestive heart failure, cardiac arrhythmias, valvular disease, peripheral vascular disease, hypertension, other neurological diseases, chronic pulmonary disease, liver disease, renal failure, AIDS, lymphoma, metastatic cancer, solid tumor, obesity, fluid and electrolyte disorders, alcohol abuse, drug abuse, and depression except for the subgroup variable.

SOFA, sequential organ failure assessment; AIDS, acquired immune deficiency syndrome; HR, hazard ratio; CI, confidence interval.

## Discussion

The present study explored the association between admission glucose level and clinical outcomes among critical sepsis patients with diabetes and found that the risk of 28-day mortality was not substantially increased in sepsis patients with diabetes who had an admission glucose level of ≥240 mg/dl compared with those who had an admission glucose level of <110 mg/dl; notably, the 28-day mortality rate was markedly reduced in the 110–240 mg/dl group (HR=0.74, 95% CI: 0.59–0.93). Furthermore, an elevated admission glucose level was significantly associated with a reduction in the 28-day mortality rate in the SOFA score ≥10 subgroup, which may imply that patients with serious conditions require a higher energy supply.

Currently, a number of studies have evaluated the glycemic control goals in sepsis patients; the Surviving Sepsis Campaign similarly recommended a glycemic level of 8–10 mmol/L in glycemic management (18). To our knowledge, only a very few studies have investigated the effect of glucose on prognosis in critical sepsis patients with diabetes. A recent study by Zohar et al. included 1,527 patients with community-onset sepsis and found that admission hyperglycemia (>200 mg/dl) correlated with increased in-hospital mortality, 30-day mortality, and 90-day mortality; moreover, this adverse outcomes were more prevalent in patients with diabetes (19). In a study of 1,059 sepsis patients, Vught et al. similarly found that severe hyperglycemia (>200 mg/dl) upon admission did not increase the 30-day mortality rate in patients with sepsis; rather, hyperglycemia was strongly associated with increased 30-, 60-, and 90-day mortality rates in patients with sepsis without diabetes (20). Moreover, Tayek et al. searched the PubMed database for publications related to sepsis, diabetes, glycemia, and prognosis; nine studies were analyzed, which reported that hyperglycemia was not related to poor outcome in sepsis patients with diabetes; the opposite was true in hyperglycemic patients without diabetes, which was an independent hazard factor for ICU and in-hospital mortality (21). Stegenga et al. examined 830 patients with severe sepsis and suggested a measurable increase in 28- and 90-day mortality rates with hyperglycemia (>200 mg/dl) compared with admission glucose at or below 200 mg/dl in sepsis patients without diabetes. Although the authors did not explicitly analyze the admission glucose level in sepsis patients with diabetes, the curve fitting plots in the article indicated that admission hyperglycemia had a relatively slight effect on 28-day mortality in sepsis patients with diabetes (22). In addition, another relatively earlier research conducted by Freire et al. demonstrated that admission hyperglycemia was not appreciably associated with in-hospital mortality (23). All of the abovementioned studies showed results similar to those of our study; that is, in sepsis patients with diabetes, admission hyperglycemia was not an independent hazard factor for poor short-term prognosis. In our study, we further revealed a non-linear relationship between admission glucose level and 28-day mortality using smoothing spline curves, with the lowest 28-day mortality in the admission glucose range of 110–240 mg/dl, which was different from those reported in other studies and was one of the highlights of our study. In the subgroup analysis, we found that higher admission glucose level was significantly associated with lower 28-day mortality rate in the SOFA ≥10 subgroup; whether this means that critically ill patients require higher energy supply deserves further investigation. These results, contrary to our common knowledge of the devastating consequences of diabetes and hyperglycemia, suggest the need for an individualized glycemic control strategy for sepsis patients with diabetes that differs from other critically ill patients since they may be able to benefit from hyperglycemia.

In the light of the available studies, however, it seems that the clinical benefit of hyperglycemia and sepsis with co-existent diabetes remains a topic that cannot be thoroughly elucidated. From the clinical point of view, diabetes can cause immune dysfunction and metabolic disorders, which inevitably induce the organism’s ability to defend against infection, in turn with catastrophic consequences. Physiologically, part of the potential mechanism can be attributed to the metabolic requirements and maintenance of the function of immune cells by glucose, with an equally critical role played by the synthetic action of immunomodulators (24, 25). Furthermore, patients with diabetes have a tolerance to hyperglycemia as a consequence of persistent high blood glucose concentrations, converting the detrimental elevated glucose into an energy reservoir (26). Additionally, the therapies administered to diabetic patients, including sulfonylureas, metformin, thiazolidinediones, and insulin, as well as the effects of diabetes on the immune system, may potentially affect the host’s response to sepsis and clinical endpoints. Therefore, further investigations are imperatively needed to comprehensively address which mechanisms contribute to the overall impact of diabetes on the outcomes of sepsis.

Even with the relatively large sample size included in our study, the limitations should not be overlooked. First, we did not account for the effect of diabetes type and diabetes medications like insulin and metformin; thus, we were unable to assess whether medications and diabetes type have an effect on outcomes at this point. Next, we cannot exclude the possibility of new-onset diabetes since data on HbA1c levels are not available. Moreover, we were not able to obtain information about the duration and severity of diabetes; thus, it was impossible to measure the effect of these factors on the outcome as well. Third, we used the first blood glucose measurement obtained after admission to the ICU for the purpose of eliminating the effect of medical therapies in the ICU, and the results were different from those of studies investigating glycemic control, although our results may help identify appropriate glycemic control strategies to some extent. Finally, we should interpret these results with caution, as the association analysis should not be mistaken for causality. Therefore, further in-depth basic and clinical studies are warranted to enrich the category of findings.

## Conclusion

Admission hyperglycemia was not a risk factor for short-term prognosis in critical ill sepsis patients with diabetes; rather, a lower blood glucose level was associated with increased risk of poor prognosis. Notably, an elevated admission glucose level was significantly associated with a reduction in 28-day mortality rate in the SOFA score ≥10 subgroup; whether this implies that patients with severe illness require a higher energy supply deserves further research.

## Data availability statement

Publicly available datasets were analyzed in this study. This data can be found here: The raw data itself is from a third-party dataset available from MIMIC-III, a freely accessible critical care database. Reproduction of their data is not permitted according to the Data Use Agreement of the database but access can be requested here: https://mimic.physionet.org/gettingstarted/access.

## Ethics statement

The access of the database has been approved by the institutional review boards of both Beth Israel Deaconess Medical Center and Massachusetts Institute of Technology Affiliates (Record ID: 33460949). Written informed consent for participation was not required for this study in accordance with the national legislation and the institutional requirements.

## Author contributions

SL and DL designed the study and wrote the draft of this manuscript, WH mainly performed data extraction and statistical analysis, SL revised this manuscript. All authors are involved in data correction. Written consent to publication was obtained.
